# High serum CRP influences myocardial miRNA profiles in ischemia-reperfusion injury of rat heart

**DOI:** 10.1371/journal.pone.0216610

**Published:** 2019-05-07

**Authors:** Eun Na Kim, Chong Jai Kim, So Ra Kim, Jung-A. Song, Han Choe, Ki-Bong Kim, Jae-Sung Choi, Se Jin Oh

**Affiliations:** 1 Department of Pathology, University of Ulsan College of Medicine, Asan Medical Center, Seoul, Republic of Korea; 2 Asan Laboratory of Perinatal Science, Asan Medical Center, Seoul, Republic of Korea; 3 Department of Physiology, Asan-Minnesota Institute for Innovating Transplantation, Bio-Medical Institute of Technology, University of Ulsan College of Medicine, Asan Medical Center, Seoul, Republic of Korea; 4 Department of Thoracic and Cardiovascular Surgery, Seoul National University College of Medicine, Seoul National University Hospital, Seoul, Republic of Korea; 5 Department of Thoracic and Cardiovascular Surgery, Seoul National University College of Medicine, SMG-SNU Boramae Medical Center, Seoul, Republic of Korea; Indiana University School of Medicine, UNITED STATES

## Abstract

**Objective:**

Prognosis of myocardial infarction tends to be worse when serum C-reactive protein (CRP) level is high. miRNAs are also known to be involved in different pathogeneses of heart diseases such as myocardial infarction. However, how CRP is involved in myocardial infarction has not been fully elucidated. We hypothesized that serum CRP changes the miRNA profile during ischemia-reperfusion injury (IRI) of the myocardium. To confirm this hypothesis, we performed global miRNA expression profiling of myocardium using IRI and CRP infusion rat model.

**Methods:**

After ligation of the coronary artery of rat hearts, human serum CRP was intravenously injected, and reperfusion was performed (I/R+CRP group, n = 6). Control group consisted of the sham group (n = 3), IV CRP infusion group (CRP only, n = 3), and the I/R-only group (I/R only, n = 5). We evaluated 423 miRNA expression in non-ischemic areas and areas at risk (AAR) of each group using NanoString nCounter miRNA expression assay.

**Results:**

MiR-124 was downregulated in non-ischemic myocardium in CRP-only group. In AAR, 7 miRNAs were commonly upregulated in both I/R-only and I/R+CRP groups. And additional 6 miRNAs were upregulated in the I/R+CRP group (miR-33, miR-409-3p, miR-384-3p, miR-3562, miR-101a, and miR-340-5p). Similarly, in the non-ischemic areas, 6 miRNAs were commonly upregulated in both I/R-only and I/R+CRP groups, and additional 5 miRNAs changed in the I/R+CRP group (upregulation of miR-3559-5p, miR-499, and miR-21 and downregulation of miR-500 and miR-532-3p).

**Conclusion:**

We showed that when serum CRP level is high, IRI results in multiple miRNA profile changes not only in ischemic areas but also in non-ischemic myocardium. Our results may provide a strong basis for studying the role of CRP and miRNAs in ischemic heart disease.

## Introduction

C-reactive protein (CRP) is an acute reactant protein that is rapidly released from the liver when induced by interleukin-6 in the presence of inflammation, cell injury, or infection [1, 2]. Serum CRP level increases during acute myocardial infarction and is considered a valuable prognostic marker of ischemic heart disease [3–6].

CRP is present in the blood as a pentamer, and when it encounters damaged cell membranes, pentameric CRP (pCRP) undergoes a structural change into a monomeric form and is deposited in the damaged site [7–9]. Although pCRP plays an anti-inflammatory role in the blood, monomeric CRP (mCRP) deposited in the damaged tissue activates the complement cascades, monocytes, and oxidative damage by reactive oxygen species formation, resulting in aggravation of cell damage [10–14]. Thiele et al demonstrated that mCRP had a pathogenic role in myocardial infarction [15]. Accordingly, our previous study showed that mCRP is deposited in areas at risk (AAR) of the myocardium (ischemic, but not infarcted) with ischemia-reperfusion injury in rat heart [16].

microRNAs (miRNAs) are small non-coding RNAs that attach to the 3’ untranslated region of target mRNAs and negatively regulate gene expression by leading to mRNA degradation or translational repression [17–20]. miRNAs are involved in cell development, proliferation, differentiation, apoptosis, metabolism, and determining the fate of cells. Therefore, changes in miRNAs are deeply associated with the pathophysiology of many diseases [21, 22]. In particular, miRNAs play an essential role in the development of various cardiovascular diseases and are used as a marker of progression and prognosis of the disease [23, 24].

However, there has been no study about the relation of two critical factors in ischemic heart disease: serum CRP and myocardial miRNAs. In the present study, we hypothesized that serum CRP would change miRNA profiles of damaged myocardium by ischemia-reperfusion injury. To prove this hypothesis, we performed myocardial miRNA expression profiling in the ischemia-reperfusion injury rat model with CRP infusion using high-throughput NanoString nCounter technology.

## Materials and methods

### Removal of sodium azide from human CRP

Sodium azide in commercial CRP preparations, which is obtained from human plasma (C4063; Sigma-Aldrich, Saint Louis, Missouri), was found to induce proapoptotic effects in human umbilical vein endothelial cells [25, 26]. Therefore, we carefully dialyzed out in CRP preparations in a large volume of Tris-HCL buffer.

### Endotoxin assay of human CRP

We verified that commercial CRP (C4063) does not contain lipopolysaccharide (LPS). The amount of the endotoxin was determined using a quantitative test, specifically the endpoint chromogenic Limulus amebocyte lysate (LAL) test (Lonza, Basel, Switzerland). Fifty microliters of a 1 μg/mL sample, or an endotoxin standard (2.0, 1.0, 0.5, 0.25, and 0.1 EU/mL), were dispensed into prewarmed 37°C endotoxin-free microplate wells. In each well, 50 μL of LAL reagent was added. Wells were incubated at 37°C for 10 minutes. After incubation, 100 μL of prewarmed chromogenic substrate solution was added to each well and incubated at 37°C for 6 minutes. Next, 100 μL of stop reagent [25% (v/v) acetic acid] was added and analyzed at 405 nm using a spectrophotometer. After analyzing the absorbance, the units of endotoxin were calculated using the standard curve obtained from the standard solution [15]. The extrapolated endotoxin level of the CRP protein sample after the dialysis was 0.129 EU/mL. The detection limit on the low side was 0.131 EU/mL. The endotoxin level of the CRP sample was below the detection limit.

### CRP pentamer report

A previous study reported that a commercially obtained source of CRP contained measurable quantities of mCRP [27]. Therefore, we confirmed that a commercially available CRP (C4063; Sigma-Aldrich) was pentameric, spontaneously modified to monomeric form, or not contaminated with mCRP. The mCRP was obtained by the treatment of dialyzed CRP with 8 M urea and 10 mM EDTA at 37°C for 1 hour. 0.9 μg of pCRP and mCRP was loaded onto 10% glycine gel containing 1/20th of normal levels of sodium dodecyl sulfate (0.005%). The protein bands were visualized using Coomassie staining, and the identity of the CRP was confirmed using Western blotting with anti-CRP Ab (ab32412; Abcam, Cambridge, United Kingdom; 1:250 dilution), which detects both pCRP and mCRP [15, 27].

**Fig 1:**
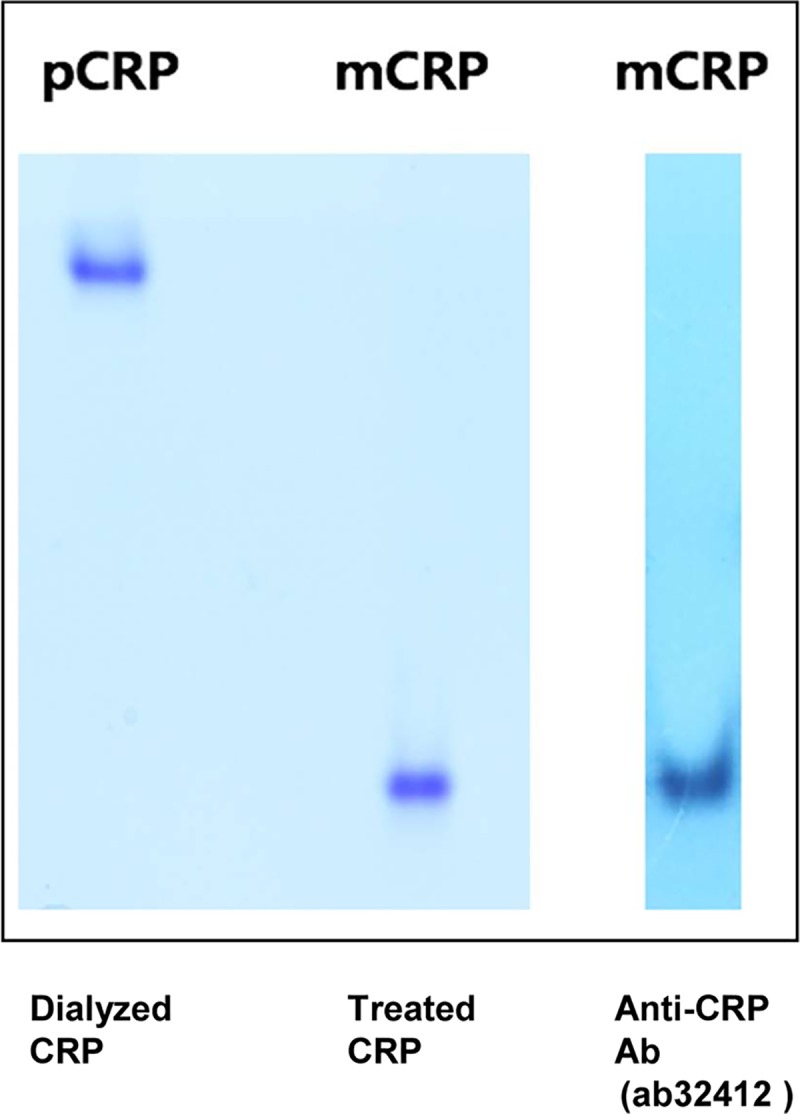
Western blot of dialyzed CRP and treated CRP with urea and EDTA. The dialyzed CRP appeared at much higher sizes than the treated CRP did, suggesting pentameric CRP (pCRP) and monomeric CRP (mCRP), respectively. Western blotting showed that the mCRP was detected with the anti-CRP antibody (ab32412). pCRP, but no mCRP, was found in infused human CRP.

The dialyzed CRP appeared at much higher sizes than the treated CRP did, suggesting pCRP and mCRP, respectively. The dialyzed CRP did not show any bands that were monomer size. The mCRP was detected with the anti-CRP antibody (ab32412; Abcam; 1:250 dilution). The pCRP was not transferred well onto the membrane. Therefore, the dialyzed CRP was shown to be in pentameric form (Fig 1).

### Animals

We constructed a myocardial ischemia-reperfusion injury model using female Sprague Dawley rats weighing between 220 g and 260 g (gestational age range, 12–14 weeks). The animals were treated in compliance with the Guide for the Care and Use of Laboratory Animals (National Academy of Sciences, Washington, DC). Animal use protocols were approved by the Institutional Animal Care and Use Committee (IACUC) at the SMG-SNU Boramae Medical Center Biomedical Research Institute (approval number: 2016–0043). Anesthesia was performed via inhalation of isoflurane (4%) for induction followed by the intraperitoneal administration of Zoletil 50 (Virbac, Carros, France; 0.12 ml) and Xylazine (Rompun 2%; Bayer Korea, Gyeonggi-do, Republic of Korea; 0.02 ml) for maintenance. The rats were intubated with cannulas (16-gauge intravenous catheter, REF 382457; BD, Sandy, Utah) and connected to ventilators (683 rodent ventilator; Harvard Apparatus, Holliston, Massachusetts). Positive-pressure ventilation using room air at a tidal volume of 2.5 ml to 3.0 ml (10 ml/kg) and 63 to 67 breaths/min was maintained to prevent atelectasis during the procedure. Left thoracotomy via the fifth intercostal space was performed, and the pericardium was opened to expose the left coronary artery.

### Experimental protocols

**Fig 2:**
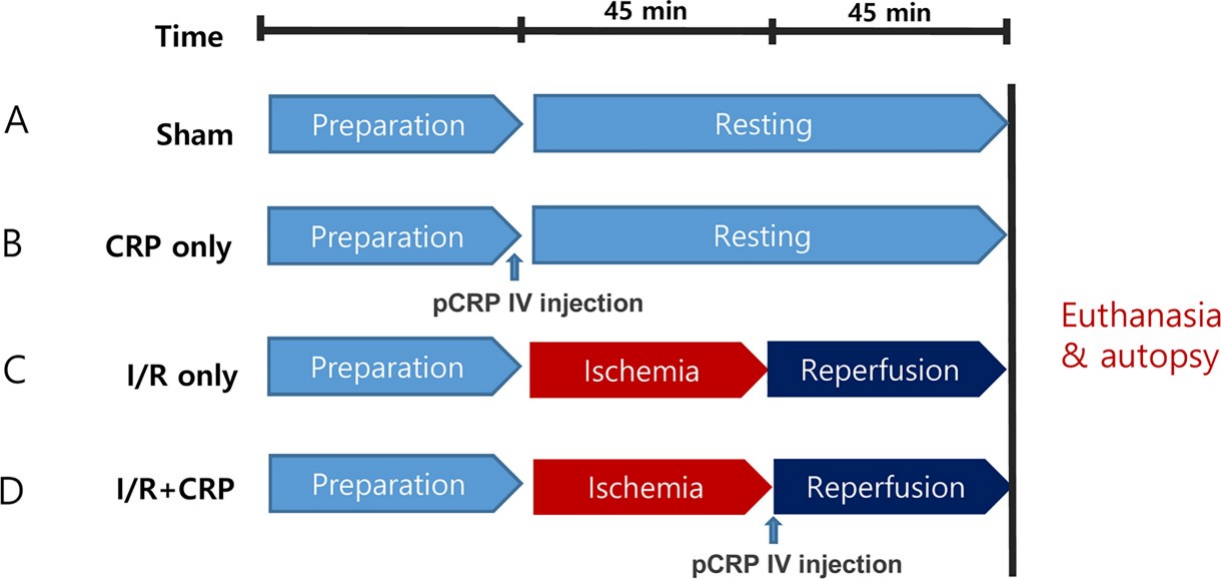
Schematic illustration of experimental protocols. (A) Sham group. (B) CRP-only group. (C) I/R-only group. D, I/R+CRP group.

The experimental group consisted of the sham group (n = 3), CRP-only group (n = 3), I/R-only group (n = 5), and the I/R+CRP group (n = 6). On the sham group (n = 3), we performed left thoracotomy and pericardiotomy without manipulation of the heart. After carrying out the procedures and then waiting for 90 minutes, we euthanized the rats and then performed autopsies (Fig 2A). On the CRP-only group (n = 3), we performed left thoracotomy and pericardiotomy and infused pCRP via the femoral vein and then waited for 90 minutes without manipulation of the heart. After that, we euthanized the rats and performed autopsies (Fig 2B). On the I/R-only group (n = 5), ischemia-reperfusion injury (IRI) was induced by ligating the left anterior descending (LAD) coronary artery approximately 2 mm distal to its origin by snaring it with 6–0 nylon double sutures (buttressed with a small piece of plastic tube for 45 minutes) and reperfusion for 45 minutes. After reperfusion, the heart was quickly excised (Fig 2C). For the I/R+CRP group (n = 6), 100 μg of highly purified and dialyzed human CRP was infused via the femoral vein after the release of coronary ligation (Fig 2D).

### Evans blue and 2,3,5-triphenyltetrazolium chloride staining to determine infarct areas, areas at risk, and non-ischemic areas

One milliliter of diluted heparin solution (2500 IU/mL) was infused via the coronary ostium while the ascending aorta was clamped. The LAD, which had been occluded for infarct production, was again occluded with a 6–0 nylon suture, and 1% Evans blue was injected to stain the perfused myocardium. The AAR and infarct (whole ischemic areas) were left unstained. Non-ischemic areas were stained deep blue.

**Fig 3:**
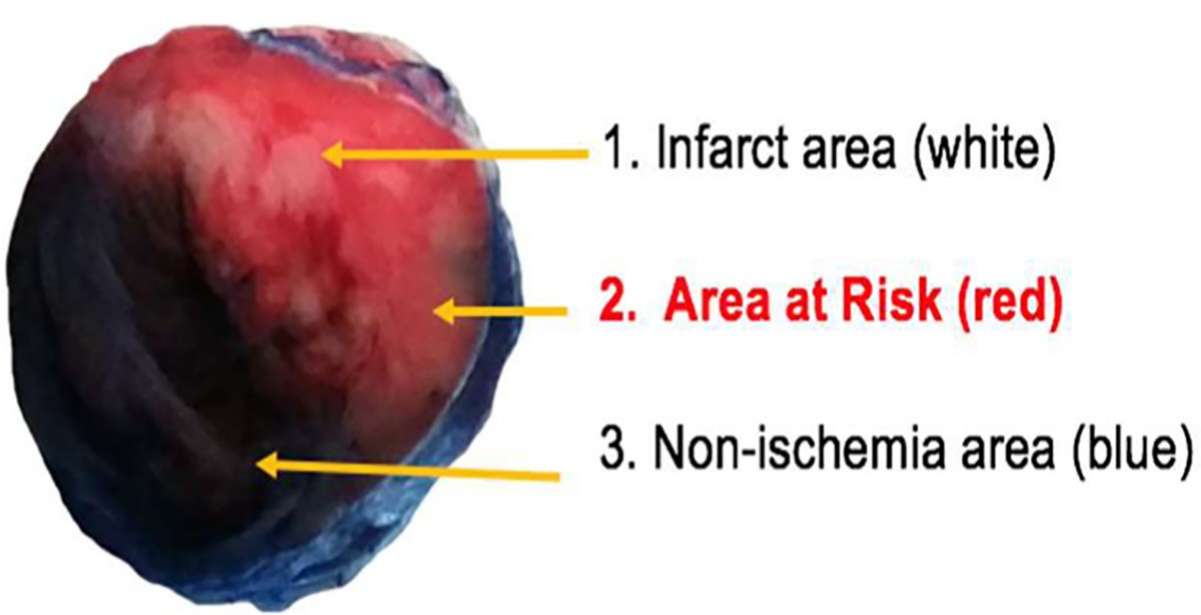
Evans blue and 2,3,5-triphenyltetrazolium chloride (TTC) staining. Evans blue and TTC staining determined infarct area (1, white), area at risk (2, red), and non-ischemic area (3, blue).

The left ventricles were cut from the apex to the base in 4 transverse slices. For the sliced sections of the myocardium, the middle parts of the left ventricle at the same cross section were used to measure the ischemia and infarct sizes. Selected middle portions were then cut into 4 mm slices, 1 of which was incubated in TTC (Sigma Chemical) that had been dissolved in a 100 mmol/L phosphate buffer for 15 minutes. TTC stains active mitochondria pink and negatively stains infarcted areas. Therefore, the infarcted zones remained white, whereas the viable areas (non-ischemic areas and AAR) were stained deep red with TTC. After Evans blue and TTC staining, the non-ischemic areas were stained blue, AAR were stained red, and the infarct was stained white (Fig 3). The fresh tissues were dissected by color, separated, and stored at -70°C for miRNA isolation. After Evans blue and TTC staining, fresh myocardium tissues (about 2 × 2 × 2 mm^3^) were excised according to their color: blue (non-ischemic area), red (AAR, ischemic but viable area), and white (infarcted area). In the sham group and the CRP-only group, only the blue myocardium was sampled because LAD ligation was not performed in them. Excised myocardium tissues were immediately snap-frozen at -70°C in order to avoid RNA degradation.

### RNA extraction and quantification

Total RNA was isolated from fresh frozen myocardial tissues dissected from the blue (non-ischemic area), red (AAR), and white (infarcted area) zones. Frozen tissue samples were processed on ice to prevent thawing; 3 mg of tissue was added to 700 μl QIAzol Lysis Reagent (QIAGEN, CA, USA) and homogenized using taco Prep Bead Beater (Genereach, Taichung City, Taiwan). After resting the homogenate at room temperature for 5 minutes, 140 μl chloroform was added and centrifuged at 12,000 × g for 15 min at 4°C. RNA extraction was performed using the miRNeasy Mini Kit (QIAGEN) following the manufacturer’s instructions. RNA yield and purity were assessed using the DS 11 Spectrophotometer (Denovix Inc, DE, USA).

### miRNA microarray expression by NanoString nCounter platform

NanoString rat panel expression levels of 423 different miRNAs were measured using 100 ng of miRNA and the nCounter Rat miR version 1.5 Expression Assay Kit (NanoString Technologies, Seattle, Washington). The assay incorporates 420 mature miRNAs based on miRBase version 17 and includes 6 positive mRNA controls, 8 negative mRNA controls, 3 ligations positive, 3 ligation negative synthetic miRNA controls, and 4 miRNA housekeeping controls (internal reference genes; Actb, B2m, Gapdh, and Rpl19).

Hybridizations were carried out by combining 5 ul of each RNA sample with 20 ul of nCounter Reporter probes in hybridization buffer and 5 ul of nCounter Capture probes (for a total reaction volume of 30 ul) overnight at 65°C for 16 to 20 hours. Excess probes were removed using two-step magnetic bead-based purification on the nCounter Prep Station (NanoString Technologies). Abundances of specific target molecules were quantified on the nCounter Digital Analyzer by counting the individual fluorescent barcodes and assessing the target molecules. For each assay, a high-density scan encompassing 280 fields of view was performed. The data was collected using the nCounter Digital Analyzer after taking images of the immobilized fluorescent reporters in the sample cartridge with a CCD camera.

### Data analysis

miRNA data analysis was performed using the nSolver Analysis Software (version 3.0) freely available from NanoString Technologies. The global mean of top 100 miRNA expressers in the samples tested was used to content normalize the miRNA expression ac all samples as described previously [28, 29].

Statistical analyses were performed with the R software [30]. Thirty miRNAs with average expression count > 10 were selected. Fold change > 2 was set as upregulation, and < -2 as downregulation. P values < .05 were considered statistically significant.

## Results

We compared the expression level of 420 rat miRNAs in non-ischemic myocardium (blue area) and ischemic but viable myocardium (red area) of each group (CRP-only group, I/R-only group, and I/R+CRP group) to that of the sham group. Normalized NanoString signals data are provided in supporting information files (S1 and S2 Data). For the CRP-only group, we infused only CRP without ischemia-reperfusion injury. Therefore, we could obtain only non-ischemic myocardium in the CRP-only group. The level of miR-124 was significantly lower in the CRP-only group than the sham group (fold change, -3.07; P = .02).

**Fig 4:**
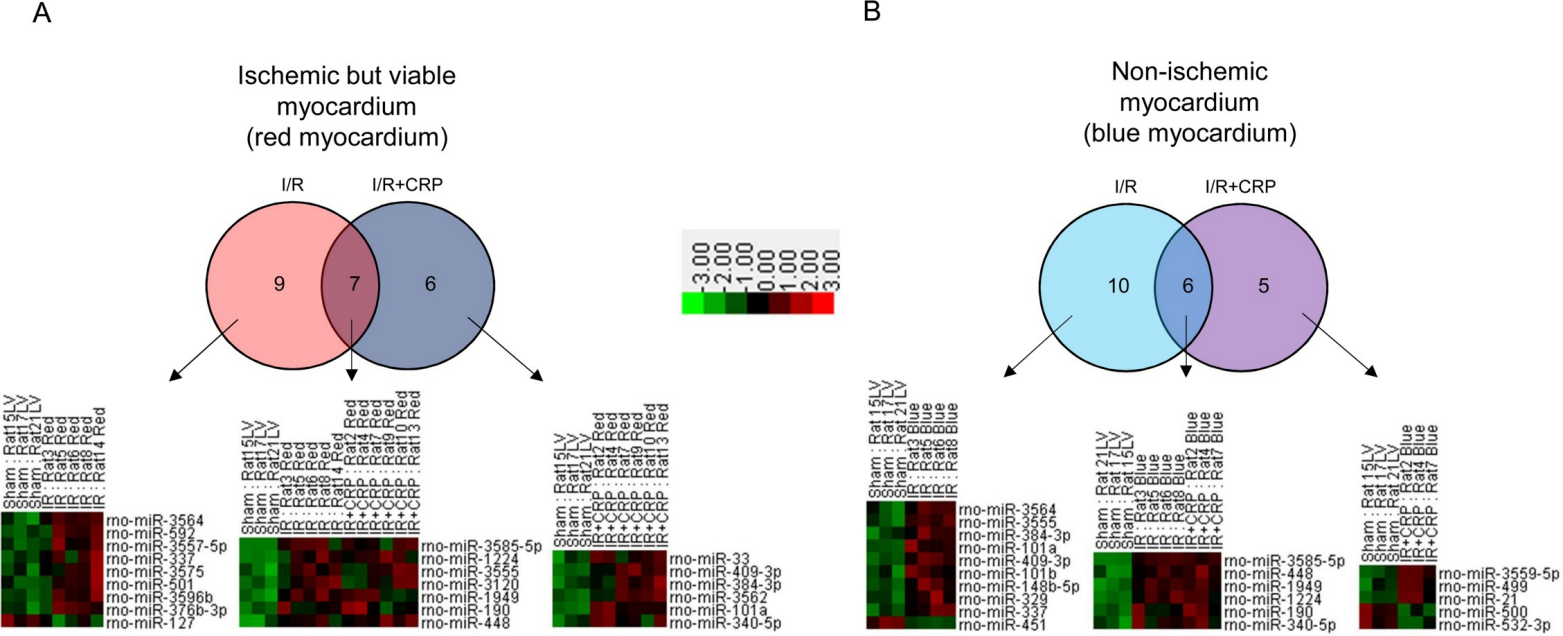
miRNA expression profiles. Global miRNA expression analysis of ischemic but viable myocardium (A, red myocardium by Evans blue and TTC staining) and non-ischemic myocardium (B, blue myocardium by Evans blue and TTC staining). Venn diagrams (upper) and corresponding heat maps (lower) show significantly different expressions of miRNAs when compared to sham group. miRNAs with average expression count > 10, fold change >2 or < -2, and P value < .05 were selected.

For the ischemic but viable myocardium (red myocardium), 8 miRNAs (miR-3564, miR-592, miR-3557-5p, miR-337, miR-3575, miR-501, miR-3596b, and miR-376b-3p) were upregulated, and 1 miRNA (miR-127) was downregulated in the I/R-only group. Seven miRNAs (miR-3585-5p, miR-1224, miR-3555, miR-3120, miR-1949, miR-190, and miR-448) were commonly upregulated in both the I/R-only group and the I/R+CRP group. Similar to the trend of ischemic myocardium, additional 6 miRNAs (miR-33, miR-409-3p, miR-384-3p, miR-3562, miR-101a, and miR-340-5p) were upregulated only in non-ischemic myocardium in the I/R+CRP group (Fig 4A; Table 1).

**Table 1:** Genes significantly changed in each group compared to the sham group in ischemic but viable myocardium (red area by evans blue and TTC staining).

Gene Name I/R vs Sham	Fold change	P value	Average expression count		Gene Name I/R+CRP vs Sham	Fold change	P value	Average expression count	
			I/R	Sham				I/R+CRP	Sham
rno-miR-3585-5p	31.6	.00484081	31.6	1	rno-miR-3585-5p	20.96	.00199687	20.96	1
rno-miR-1224	3.62	.00659538	97.09	26.85	rno-miR-1224	3.93	.00052359	105.43	26.85
rno-miR-3555	3.18	.01800967	12.44	3.92	rno-miR-3555	3.09	.01780053	12.1	3.92
rno-miR-3120	2.7	.00485023	13.78	5.11	rno-miR-3120	2.16	.01399395	11.06	5.11
rno-miR-1949	2.58	.00168606	236.52	91.68	rno-miR-1949	2.17	.00439291	198.75	91.68
rno-miR-190	2.53	.01061211	49.88	19.68	rno-miR-190	2.84	.01642446	55.82	19.68
rno-miR-448	2.49	.02966429	15.06	6.04	rno-miR-448	3.2	.01399491	19.3	6.04
rno-miR-3564	8.56	.03530545	31.15	3.64	rno-miR-33	4.97	.03085913	10.72	2.16
rno-miR-592	3.46	.01995102	10.76	3.11	rno-miR-409-3p	3.96	.00059162	13.56	3.42
rno-miR-3557-5p	2.56	.02299442	15.55	6.08	rno-miR-384-3p	3.82	.01736719	13.06	3.42
rno-miR-337	2.28	.04114917	14.07	6.16	rno-miR-3562	3.74	.00213201	11.87	3.18
rno-miR-3575	2.27	.04284919	25.34	11.16	rno-miR-101a	2.59	.00006868	26.33	10.17
rno-miR-501	2.2	.04875308	63.05	28.67	rno-miR-340-5p	2.03	.04336423	23.46	11.55
rno-miR-3596b	2.15	.02778967	20.3	9.44					
rno-miR-376b-3p	2.06	.04421247	10.4	5.04					
rno-miR-127	-2.68	.02952817	4.44	11.9					

In non-ischemic myocardium (blue area), 9 miRNAs were upregulated, and 1 miRNA (miR-451) was downregulated in the I/R-only group. Six miRNAs (miR-3585-5p, miR-448, miR-1949, miR-1224, miR-190, and miR-340-5p) were commonly upregulated in both the I/R-only and I/R+CRP groups than in the sham group. However, unlike in the CRP-only and I/R-only groups, 3 miRNAs (miR-3559-5p, miR-499, and miR-21) were upregulated, and 2 miRNAs (miR-500 and miR-532-3p) were downregulated in only the I/R+CRP group (Fig 4B; Table 2).

**Table 2:** Genes significantly changed in each group compared to the sham group in non-ischemic myocardium (blue area by evans blue and TTC staining).

Gene Name I/R vs Sham	Fold change	P value	Average expression count		Gene Name I/R+CRP vs Sham	Fold change	P value	Average expression count	
			I/R	Sham				I/R+CRP	Sham
rno-miR-3585-5p	56.94	.00034394	61.73	1.08	rno-miR-3585-5p	50.87	.00132952	55.16	1.08
rno-miR-448	3.16	.01663447	23.42	7.4	rno-miR-448	4.25	.01132464	31.46	7.4
rno-miR-1949	3	.00115536	336.69	112.32	rno-miR-1949	3.03	.00264767	340.33	112.32
rno-miR-1224	2.89	.004928	95.2	32.9	rno-miR-1224	2.75	.02371731	90.48	32.9
rno-miR-190	2.84	.0367035	68.39	24.11	rno-miR-190	4.63	.037218	111.6	24.11
rno-miR-340-5p	2.23	.03751136	31.51	14.15	rno-miR-340-5p	2.42	.04547073	34.25	14.15
rno-miR-3564	6.74	.0372734	30.03	4.46	rno-miR-3559-5p	4.12	.00431564	12.72	3.09
rno-miR-3555	3.73	.01814172	17.9	4.8	rno-miR-499	2.22	.04658707	10329.86	4660.75
rno-miR-384-3p	3.55	.02377503	14.89	4.19	rno-miR-21	2.06	.04408989	718.04	348.35
rno-miR-101a	3.35	.01129538	41.7	12.46	rno-miR-500	-2.13	.02676364	4.97	10.61
rno-miR-409-3p	2.94	.00162202	12.33	4.19	rno-miR-532-3p	-2.19	.03442331	20.53	45.01
rno-miR-101b	2.64	.036637	21.75	8.24					
rno-miR-148b-5p	2.46	.01162151	18.06	7.36					
rno-miR-329	2.3	.02992854	16.76	7.29					
rno-miR-337	2.24	.01032434	16.89	7.55					
rno-miR-451	-6.46	.0135633	9.5	61.4					

## Discussion

This study shows that serum CRP influences the miRNA profiles in damaged myocardium by ischemia-reperfusion injury. In our experiments, the expression levels of multiple miRNAs change in non-ischemic myocardium as well as in ischemic myocardium in response to CRP infusion.

For ischemic area, 7 miRNAs upregulated in both I/R-only and I/R+CRP group are regarded as changes induced by IRI. Six miRNAs upregulated only in I/R+CRP group (miR-33, miR-409-3p, miR-384-3p, miR-3562, miR-101a, and miR-340-5p) are considered to have been influenced by CRP infusion (Fig 4A; Table 1). Four of these 6 miRNAs identified only in the I/R+CRP group (miR-33, miR-409-3p, miR-384-3p and miR-101a) were found to be involved in myocardial disease. Recent studies have shown that the expression level of miR-33 is elevated in heart failure [31] and that miR-33 promotes myocardial fibrosis [32] and vascular atherosclerosis [33]. Currently, miR-33 is being considered as a new therapeutic target [34]. Consequently, our study suggests that upregulation of miR-33 may be a missing link between bad prognosis of ischemic heart disease and high serum CRP level. Interestingly, the miRNAs (miR-409-3p, miR-384-3p, and miR-101a) that were upregulated after CRP infusion in our present study have been shown to have cardioprotective roles or downregulated in patients with heart diseases: miR-409-3p was significantly downregulated in mitral regurgitation patients with heart failures in a previous case-control study [35]; miR-384-3p was downregulated in ischemic myocardium of rat and was shown to regulate apoptosis pathway to play a cardioprotective role in myocardial ischemia [36]; miR-101a was shown to mitigate cardiac fibrosis in postinfarct rats [37]; miR-340-5p was shown to inhibit cell proliferation and increase apoptosis in various types of cancer cells [38–40].

For non-ischemic myocardium, 6 miRNAs were changed in both I/R-only and I/R+CRP groups, and additional 5 miRNAs (upregulation of miR-3559-5p, miR-499, miR-21 and downregulation of miR-500 and miR-532-3p) were changed in I/R+CRP group; this is regarded as the effect of CRP infusion (Fig 4B; Table 2). Among these miRNAs, miR-499, miR-21, and miR-500 are known to be involved in heart disease. miR-499 is highly expressed in myocardiocytes and increase in acute myocardial infarction [41–45]. miR-21 has an important role in cardiac fibrosis after myocardial infarction [46] and for regulating fibroblast proliferation [47]. Furthermore, miR-500 was downregulated in patients with diastolic dysfunction [48].

Functional recovery after ischemic injury depends on the amount of remaining non-ischemic myocardium and the recovery from reperfusion of the ischemic area. Many miRNAs change both in non-ischemic and ischemic myocardium when IRI occurs in high serum, and the changes may have important roles in aggravating heart conditions. The pathogenesis of dysregulation of these miRNAs and its role should be elucidated in future research.

### miR-124 is downregulated in non-ischemic myocardium with high serum CRP level

Interestingly, miR-124 is significantly downregulated in CRP-infused myocardium even in the absence of ischemic injury. Previous studies have shown the harmful effect of miR-124 on the myocardium. miR-124 expression is known to be an adverse prognostic factor in heart failure that suppresses angiogenesis [49]. Downregulation of miR-124 is known to have a protective role in damaged myocardium, suppressing Angiotensin II–induced myocardial hypertrophy along with the attenuation of ER stress [50]. A substudy of a randomized clinical trial of 519 patients showed that miR-124-3p level was associated with neurological outcomes and lower survival rates in cardiac arrest patients [51]. Our results showed that serum CRP increased miR-124 level. We suggest that an increase in miR-124 level induced by CRP might be associated with bad prognosis of cardiovascular diseases.

### Infusion of dialyzed human pentameric CRP in rat ischemia-reperfusion injury model

Whereas the serum CRP released from rat liver does not activate the complement cascade by itself, human CRP does cause the complement cascade in a rat, mimicking the effects of CRP on complement activation in humans [52, 53]. Therefore, many studies that focus on the effects of CRP on complement used human CRP instead of rat CRP. Therefore, the present study used human CRP and not rat CRP.

To obtain purified endotoxin-free, azide-free human CRP, we carefully dialyzed and removed sodium azide from commercial CRP obtained from human plasma. A previous study showed that LPS contamination in commercial CRP was responsible for cell activation events [26]. We then performed endotoxin assay to confirm the absence of LPS in the infused human CRP. We also confirmed whether the infused CRP had spontaneously changed to a monomeric form. Using these methods, we were able to conduct a sophisticated, well-controlled experiment.

### Polymeric CRP and monomeric CRP

We performed immunohistochemical staining of heart sections using formalin-fixed, paraffin-embedded, 4-μm-thick tissue sections with a human monoclonal anti-CRP antibody (C1688; Sigma-Aldrich; 1:400 dilution), which specifically detects the monomeric form of CRP (mCRP). The I/R+CRP group showed diffuse and strong immunopositivity for anti-mCRP antibody in their damaged myocardium (data not shown), which is in line with our previous finding that mCRP is deposited on AAR myocardium damaged by IRI [16]. Moreover, CRP mRNA was not detected by PCR in our previous study, which supports the idea that mCRP immunopositivity is the result of mCRP deposition rather than production of CRP from the myocardium. We speculate that the deposited mCRP may carry some role in regulating the miRNA profiles in the damaged myocardium.

We showed that mCRP deposition activates complement, mitochondria destruction, autophagy production, and apoptosis in AAR myocardium and results in the increase in infarct size [16], which is consistent with the findings from a study by Yang et al. [54] mCRP deposition was limited to AAR in our previous study. In this study, miRNA profile changes occur in both AAR and non-ischemic areas after CRP injection.

### Limitations of the study

We had to select and dissect 3 areas of rat heart based on the results of Evans blue and TTC staining and therefore were only able to obtain small specimens of rat heart tissue and consequently a small amount of RNA. Only NanoString apparatuses require smaller amounts of material; we could perform only the NanoString method to examine the miRNA profiles and did not validate our findings with other methods. Nevertheless, the NanoString nCounter platform is not an enzyme-dependent method, lacks RNA-amplification steps, and performs miRNA profiling with digital precision. It is therefore accurate and less error-prone [55]. Results obtained using NanoString apparatuses do not require further validation by another method [56]. The NanoString nCounter platform is being used to validate the results of another platform, RNA-Seq [57].

We did not experiment with pCRP infusion before ischemic injury, which could reflect clinical situations involving pre-existing long-standing inflammatory processes before an ischemic event such as diabetes mellitus and severe obesity. There are reports that elevated CRP level during myocardial infarction is associated with myocardial damage and consequent elevated IL-6 level rather than pre-existing inflammation.[4, 6, 58] We also intended to find changes in miRNAs in CRP-deposited damaged myocardium by injecting serum CRP after infarction.

CRP supplementation may not accurately mimic the pathological increase in serum CRP in response to myocardial infarction, because pathologically increased CRP may interact with a variety of tissues and cells. Future study should therefore utilize CRP-knockout mice in order to evaluate the role of endogenous CRP in the respective regulation of miRNA profiles in ischemic and non-ischemic myocardium.

## Conclusion

We have shown that high serum CRP level with ischemia-reperfusion injury results in myocardial miRNA changes. We also showed that the miRNA changes occurred in both ischemic and non-ischemic myocardium. Many of these miRNA changes are involved in myocardial diseases. Our results may provide a strong basis for research on the relation between CRP and miRNAs and their role in ischemic heart disease and subsequent reperfusion therapy.

## Supporting information

S1 Data Normalized NanoString signals (counts) of blue myocardium. (XLSX)

S2 Data Normalized NanoString signals (counts) of red myocardium. (XLSX)
